# Impact of the p53 status of tumor cells on extrinsic and intrinsic apoptosis signaling

**DOI:** 10.1186/1478-811X-11-27

**Published:** 2013-04-17

**Authors:** Franziska Wachter, Michaela Grunert, Cristina Blaj, David M Weinstock, Irmela Jeremias, Harald Ehrhardt

**Affiliations:** 1 Helmholtz Zentrum München, German Research Center for Environmental Health, Marchioninistrasse 25, Munich, D-81377, Germany; 2 Department of Medical Oncology, Dana-Farber Cancer Institute, Boston, Massachusetts, USA; 3 Department of Oncology / Hematology, Dr. von Haunersches Kinderspital, Lindwurmstr 4, München, 80337, Germany; 4 Division of Neonatology, University Children’s Hospital, Perinatal Center, Ludwig-Maximilians-University Munich, Marchioninistr 15, Munich, 81377, Germany

**Keywords:** p53, Mutant p53, Extrinsic, Intrinsic, TRAIL, Doxorubicin, Apoptosis

## Abstract

**Background:**

The p53 protein is the best studied target in human cancer. For decades, p53 has been believed to act mainly as a tumor suppressor and by transcriptional regulation. Only recently, the complex and diverse function of p53 has attracted more attention. Using several molecular approaches, we studied the impact of different p53 variants on extrinsic and intrinsic apoptosis signaling.

**Results:**

We reproduced the previously published results within intrinsic apoptosis induction: while wild-type p53 promoted cell death, different p53 mutations reduced apoptosis sensitivity. The prediction of the impact of the p53 status on the extrinsic cell death induction was much more complex. The presence of p53 in tumor cell lines and primary xenograft tumor cells resulted in either augmented, unchanged or reduced cell death. The substitution of wild-type p53 by mutant p53 did not affect the extrinsic apoptosis inducing capacity.

**Conclusions:**

In summary, we have identified a non-expected impact of p53 on extrinsic cell death induction. We suggest that the impact of the p53 status of tumor cells on extrinsic apoptosis signaling should be studied in detail especially in the context of therapeutic approaches that aim to restore p53 function to facilitate cell death via the extrinsic apoptosis pathway.

## Background

The known functions of p53 are becoming increasingly complex and involvement of p53 in transcriptional control impacts many cellular functions including cell death control, cell cycle arrest, cellular senescence, DNA repair, angiogenesis, cell migration and other fundamental physiologic cellular activities including cell metabolism, autophagy, stem cell renewal, embryogenesis, innate immunity and fertility [1]. The prediction of the precise action of p53 on a specific cell and within a special context remains the focus of research activities and the impact on transcriptional control and the cell cycle is strictly context-specific [2-7]. p53 is classically viewed for its transcriptional control of pro-apoptotic proteins like DR5, Caspase-8, Bax, PUMA or NOXA, and its accumulation and activation is regulated by transcription and by a panel of post-transcriptional modifications like phosphorylation, subcellular localization and interaction with negative regulators [1].

Targeted therapies that specifically modulate a specific step of cell death induction in tumor cells represent one of the main goals of ongoing preclinical and clinical studies to improve cancer therapy. For p53, the selective modulation of mutant p53 and the variation of p53 expression levels by direct or indirect stabilization represent the most promising approaches within current studies [8,9]. In contrast to the many ongoing studies using mostly biochemical modulations, the specific impact of p53 gene alterations was investigated in a limited number of studies with respect to the general impact on intrinsic cell death induction [5,7,10-12]. Within the research activities of the extrinsic cell death induction by death inducing ligands like TRAIL, the focus was exclusively drawn to the promising approaches of p53 activation by the addition of or pre-incubation with chemotherapeutic drugs or targeted stimuli [3,4,13].

In the study presented here, the specific impact of the altered p53 status on extrinsic and intrinsic apoptosis induction was studied in detail. We used different molecular approaches as has been done i.e. for tumorigenesis in different p53 genetic backgrounds before [14,15].

## Results

Restoration of p53 functionality and activation of p53 to improve the efficacy of tumor therapy have attracted much attention within many different tumor entities and have demonstrated superior cell death induction for many combinations [3,13,16]. In contrast, the presence or activation of p53 within certain therapeutic settings was shown to be of disadvantage for the therapy efficacy in vitro and in vivo [2,17]. Here we aimed to study the impact of the p53 status itself on extrinsic and intrinsic apoptosis sensitivity.

### Generation of constructs to modulate the p53 status

To study the impact of p53 on extrinsic and intrinsic apoptosis induction after defined stimuli, we used three different approaches: I) expression of p53 in wild-type or mutant conformation in tumor cells. II) knockdown of p53 by RNA interference. III) the use of pairs of tumor cells with baseline p53 expression and with the somatic knock-out of p53. For the first experimental setting, we generated p-CDH constructs either containing p53 in wild-type conformation or with specific point mutations for lentiviral transduction. The FKBP destabilization domain tagged to overexpressed p53 conducts the proteasomal degradation. Transient expression of the transgene was achieved after adding SHIELD-1.

**Figure 1:**
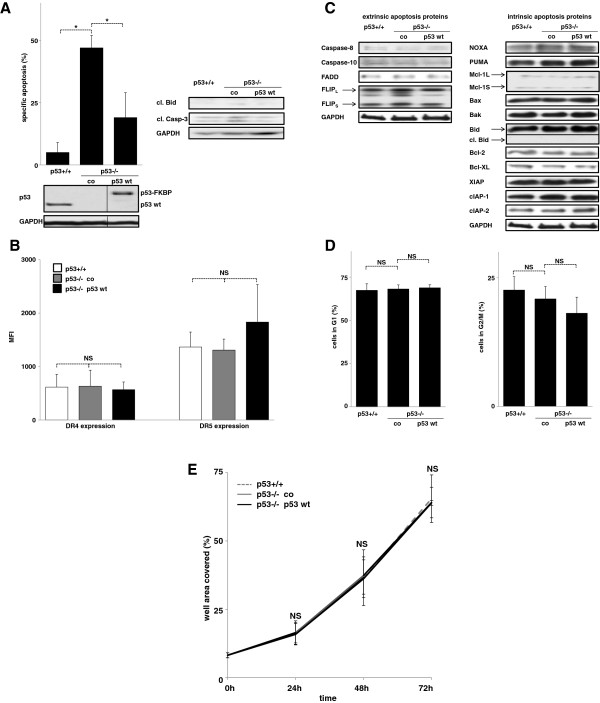
**Impact of p53 on extrinsic apoptosis sensitivity in HCT116 cells. A**) HCT116 p53+/+, untransfected HCT116 p53-/- cells (co) and HCT116 p53-/- cells transfected with pCDH p53 in wild-type conformation (p53 wt) were stimulated with TRAIL (100 ng/ml) for 48 hours. Measurements of cell death induction (left panel) and cleavage of Bid and Caspase-3 (Casp-3; right panel) are presented. Western blot analysis was performed of total cellular protein. For the ease of reading, the order of samples within the identical blot was rearranged without any further manipulation, indicated by the separating lines. cl = cleaved. **B**, **C**) Cells from Figure 1A were analyzed for TRAIL death receptor (**B**) and apoptosis signaling protein (**C**) expression. DR4 and DR5 expression was determined by FACs surface staining. The MFI (mean fluorescence intensity) was determined in the APC-Cy7 channel of a LSR II flow cytometer as described in Methods. cl. Bid = cleaved Bid. **D**, **E**) Cells from Figure 1A were analyzed for cell cycle distribution using propidium iodide staining (**D**) and for spontaneous growth by automated analyses of the well area covered over time (**E**). Cell death induction of adherent cells was measured by Nicoletti staining. Specific apoptosis was calculated as [(apoptosis of stimulated cells at end point minus apoptosis of unstimulated cells at end point) divided by (100 minus apoptosis of unstimulated cells at end point) times 100]. Statistical analysis was performed using one way RM ANOVA. *p < 0,05, NS = statistically not significant.

**Figure 2:**
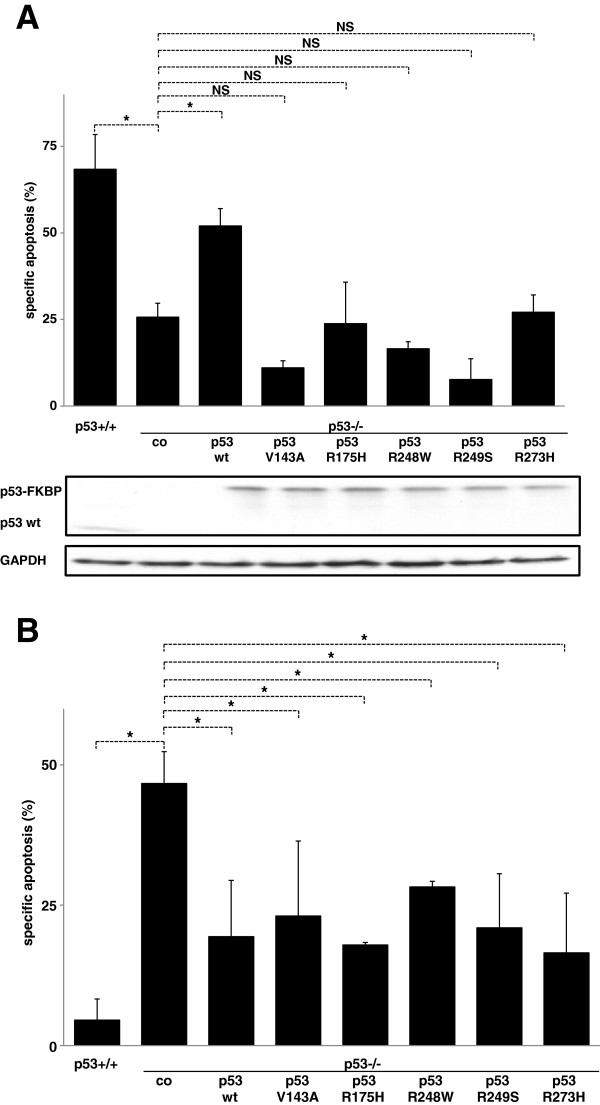
**Different impact of mutant p53 on extrinsic and intrinsic cell death induction in HCT116 cells. A**,**B**) HCT116 p53-/- cells transfected with the different pCDH constructs containing wildtype p53 or p53 with point mutation at codon 143, 175, 248, 249 or 273 were stimulated with doxorubicin (100 ng/ml, 72 hours, **A**) or TRAIL (100 ng/ml, 48 hours, **B**). Cell death induction, calculation of specific apoptosis, presentation of data and Western Blot analysis were performed as in Figure 1. Statistical analysis was performed using one way RM ANOVA. *p < 0,05, NS = statistically not significant.

**Figure 3:**
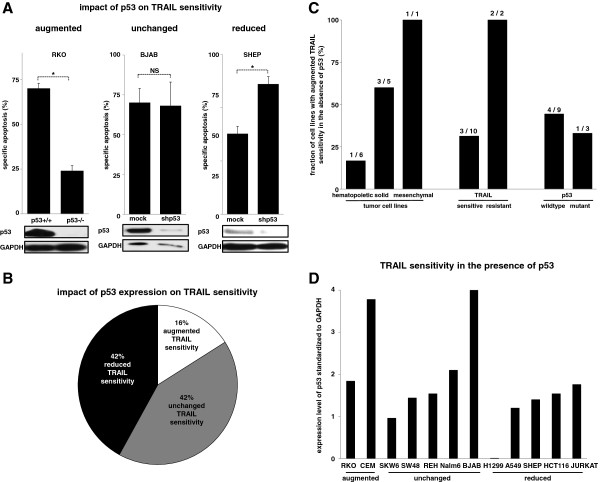
**The heterogenous impact of p53 on extrinsic cell death induction in tumor cell lines. A**) Parental RKO cells and derivative cells with somatic knockout of p53 (left panel) and BJAB (central panel) and SHEP (right panel) cells stably transfected with shRNA against p53 or a mock sequence were stimulated with TRAIL as in Figure 1A. The impact of baseline p53 expression on extrinsic cell death induction was classified as augmented cell death whenever the presence of p53 was associated with increased TRAIL sensitivity (left panel), as unchanged whenever p53 did not impact cell death by TRAIL (central panel) and as reduced when the presence of p53 diminished apoptosis induction by TRAIL (right panel). **B**) n = 12 different tumor cell lines from Figure 3A and Additional file 1: Figure S3 were classified according to their response to extrinsic cell death induction in dependence of the p53 status. **C**) Tumor cell lines from Figure 3B were categorized in the context of the tumor type, the TRAIL response of the p53 expressing cells and the p53 status of the tumor cells. TRAIL resistance was defined as cell death induction < 10%. **D**) The n = 12 cell lines from Figure 3B were analyzed for the relative p53 content as in Additional file 1: Figure S4. Samples were arranged separated for their TRAIL response in the presence of p53**.** Apoptosis induction in hematopoietic cells was determined by forwardside scatter analysis, in solid tumor cells using Nicoletti staining. Calculation of specific apoptosis, presentation of data and Western Blot analysis were performed as in Figures 1 and 2. Statistical analysis was performed using paired t-test. *p < 0,05, NS = statistically not significant.

Constructs were expressed in different tumor cells either with somatic knockout of p53 (Figures 1 and 2), loss of p53 (Additional file 1: Figure S3C) or with expression of wild-type p53 (Additional file 1: Figure S2). Five different mutant forms of p53 were introduced by mutagenesis which are known to be associated with reduced protein stability (V143A), defect of DNA binding (R248W, R273H) or structural instability (R175H, R249S) (Figure 2 and Additional file 1: Figure S2) [16]. For the second approach, the previously described technique of RNA interference against p53 using a lentiviral system was used [2-4] to generate an efficient knockdown of p53 (Figure 3A and Additional file 1: Figure S3). For the last approach, access to pairs of tumor cells either expressing p53 or with somatic knock-out of p53 was obtained (Figures 1, 2 and 3A, Additional file 1: Figure S3B).

### Impact of p53 on extrinsic apoptosis sensitivity in HCT116 cells

First we used HCT116 cells either expressing p53 in wild-type conformation or with somatic knock-out of p53. Additionally p53 in wild-type conformation was re-introduced in the p53-/- cells. To our surprise, cell death induction by the death inducing ligand TRAIL was augmented in the absence of p53 (Figure 1A). The increased apoptosis sensitivity was not attributable to any regulation of apoptosis signaling proteins involved in TRAIL signaling. On the receptor level, the p53 status did not impact DR4 and DR5 expression levels (Figure 1B). In an alternative approach, cells were transfected with the TRAIL death receptors DR4 and DR5, and overexpression did not impact apoptosis induction by TRAIL (Additional file 1: Figure S1). Downstream of the TRAIL death receptors, the p53 status did not change the expression levels of the further members of the DISC and of the most important members of the Bcl-2 and the IAP-family (Figure 1C). Beyond transcriptional control, neither the cell cycle distribution nor the spontaneous growth was affected by the p53 status (Figure 1D and E).

### Different impact of mutant p53 on extrinsic and intrinsic cell death induction

Next we studied the impact of wild-type and mutant p53 on extrinsic and intrinsic cell death induction in the HCT116 cells. As published before, wild-type p53 significantly augmented the cell death induction by doxorubicin, while the five studied p53 mutants did not affect the apoptosis inducing capacity (Figure 2A). Comparable results were obtained when doxorubicin was substituted by 5-fluorouracil, another cytotoxic drug acting by DNA damage and activation of the intrinsic apoptosis signaling cascade (data not shown) [18].

When TRAIL was studied as a classical activator of the extrinsic apoptosis signaling cascade, the impact of mutant p53 was completely different. The five different mutants of p53 reduced the TRAIL sensitivity to a similar extent as p53 in wild-type conformation further arguing against transcriptional control as the central regulatory mechanism of action (Figure 2B) [5,6]. When SHEP cells, which express p53 in wild-type conformation where overexpressed with wild-type p53 or the five different mutant forms of p53, the identical results were obtained as in HCT116 cells. While the sensitivity for doxorubicin or 5-fluorouracil was augmented when wild-type p53 was overexpressed (Additional file 1: Figure S2A and data not shown), wild-type p53 overexpression reduced the sensitivity for extrinsic apoptosis induction. As observed in HCT116 cells, not the p53 status but the overexpression of p53 was associated with reduced TRAIL sensitivity (Additional file 1: Figure S2B). Similar results were obtained, when the extrinsic signaling cascade was stimulated with the death inducing ligand FasL (data not shown).

Taken together, we have identified a distinct regulation of extrinsic and intrinsic cell death by p53. While the previously published results for the intrinsic signaling cascade were reproduced, the presence of p53 was associated with reduced extrinsic cell death induction in HCT116 and SHEP cells. Unexpectedly and in contrast to intrinsic apoptosis signaling, wild-type and mutant p53 had the identical impact on extrinsic apoptosis induction.

### The heterogenous impact of p53 on extrinsic cell death induction in tumor cell lines

To further clarify the role of p53 for extrinsic cell death, we studied n = 12 pairs of tumor cell lines expressing p53 and with downregulation / knock-out of p53. Overall, we observed three different phenotypes that were associated with the presence of p53: reduced, unchanged or augmented sensitivity for extrinsic cell death induction by TRAIL (Figure 3A and Additional file 1: Figure S3). Surprisingly, the inhibition or loss of p53 was associated with reduced TRAIL sensitivity in only 2 / 12 cell lines tested, while in 5 / 12 it did not have any impact and in 5 / 12 was associated with augmented cell death induction by TRAIL (Figure 3B). The negative action of p53 on TRAIL sensitivity was observed in hematopoietic, solid and mesenchymal tumor cells. Furthermore, the TRAIL response was independent from the TRAIL sensitivity of the parental cells and inhibition of apoptosis induction by TRAIL was detected in tumor cell lines with wildtype and mutant p53 status (Figure 3C) [16]. In line, the baseline p53 expression level varied widely between the different cell lines without any specific pattern (Figure 3D).

In summary, the impact of p53 on extrinsic cell death induction is much more complex and different actions were observed depending on the individual tumor cell which did not fit in classical categories like tumor entity, drug sensitivity or p53 mutation status of the tumor cell.

### The heterogenous impact of p53 on extrinsic cell death induction in xenografted ALL cells

**Figure 4:**
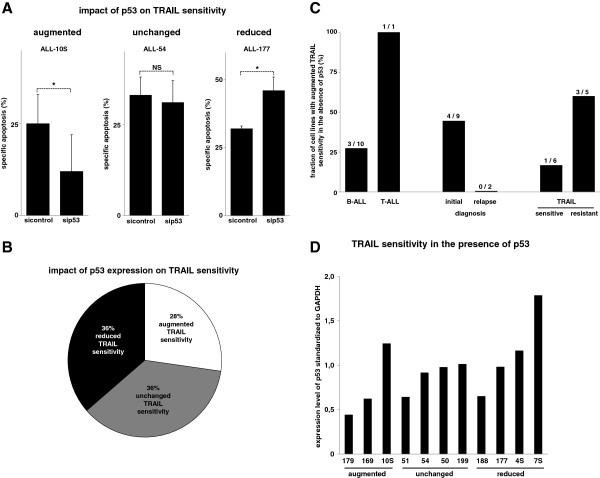
**The heterogenous impact of p53 on extrinsic cell death induction in xenografted ALL cells. A**) Patient-derived ALL-10S (left panel), ALL-54 (central panel) and ALL-177 (right panel) cells transfected with siRNA against p53 (sip53) or a control sequence (sicontrol) were stimulated with TRAIL (100 ng/ml) for 24 hours. Knockdown-efficiency of p53 by RNA interference is presented in Additional file 1: Figure S4. **B**) Cells from n = 11 different xenograft samples with wildtype p53 status (from Figure 4A and data not shown) were classified as in Figure 3B. **C**) Patient-derived ALL cells from Figure 4B were separated by the type of leukemia, the state of disease and the TRAIL sensitivity in the presence of p53 as in Figure 3C. **D**) Patient-derived ALL cells from Figure 4B were analyzed for the relative p53 content as in Figure 3D. Cell death induction, calculation of specific apoptosis, presentation of data and statistical analysis were performed as in Figure 3. *p < 0,05, paired t-test. NS = statistically not significant.

As the inhibitory effect on TRAIL sensitivity was present in nearly half of the tumor cell lines with wildtype p53 status, we chose the ALL xenograft setting to further study the relevance of wildtype p53 for inhibition of extrinsic cell death as they rarely contain p53 mutations [19,20]. We confirmed the wildtype p53 status in all ALL xenograft samples presented (data not shown). Another advantage of the employed experimental setting is that xenograft cells better resemble the in vivo situation as they do not feature non-physiologic alterations or comprise a selection of mutations that do not resemble the patient situation. We studied the impact of p53 on TRAIL sensitivity in xenografted ALL cells using the recently described experimental setting of RNA interference after amplification of primary childhood ALL cells in NOD/SCID mice [3,4,7,21-24]. RNA interference against p53 markedly reduced the p53 expression as described before (Additional file 1: Figure S4 and data not shown) [21]. As identified for the cell lines studies in Figure 3, xenografted ALL cells with knockdown of p53 reacted with three different phenotypes after stimulation with TRAIL: reduced, unchanged or augmented cell death induction (Figure 4A and data not shown). Surprisingly, the distribution of actions of p53 on extrinsic cell death was nearly identical as in the cell lines (Figure 4B). The negative effect of p53 on TRAIL sensitivity was present in B- and T-ALL cells and was detected only in samples obtained at initial diagnosis although the small number of samples at relapse studied does not allow the conclusion that in the situation of relapse the impact of p53 on extrinsic signaling is always not negative. When samples were categorized for their TRAIL response the same observation as in cell lines was detected: the fraction of samples benefiting from the knockdown of p53 was higher in the group of TRAIL resistant samples (Figure 4C). In contrast to the cell line data, the baseline p53 expression level between the different xenograft samples was less inhomogenous and showed a similar distribution for the three categories of the observed TRAIL responses (Figure 4D).

Taken together, the data obtained in established cell lines were confirmed in patient-derived tumor cells and underline the clinical relevance of the described phenotype: heterogeneous impact of p53 on extrinsic cell death induction depending on the individual tumor cell.

## Discussion

The data presented here indicate that both overexpression of wild-type or mutant p53 in cancer cells can have negative effects on cell death induction via the extrinsic apoptosis signaling cascade. These findings need to be taken into consideration during designing therapeutic strategies intended to re-introduce p53. While we were able to reproduce the formerly described heterogeneous impact of wild-type and mutant p53 on apoptosis induction by cytotoxic drugs like doxorubicin that act via the intrinsic signaling cascade [10,11], the impact of the p53 status on the extrinsic apoptosis cascade activated by death inducing ligands like TRAIL is much more complex. Investigating a panel of tumor cell lines and xenografted primary tumor cells, we detected three different actions of p53 on extrinsic apoptosis induction and the distribution of the three phenotypes was nearly equal between cell lines and xenograft cells: Promotion of extrinsic apoptosis induction, no change of apoptosis sensitivity and inhibition of cell death in the presence of p53. Surprisingly, the presence of p53 either in wild-type or mutant conformation did not significantly impact the expression level of typically p53-regulated apoptosis proteins like Bax, PUMA or DR5, spontaneous growth or cell cycle distribution [13]. While the impact of p53 on extrinsic apoptosis signaling was so far exclusively / mainly studied for the situation of p53 activation, the data provided here clearly indicate that the p53 status of the tumor cells impacts the response to extrinsic apoptosis stimuli while classical targets of p53 accumulation are not affected. Using RNA interference for p53 knockdown, somatic knockout of p53 and p53 re-expression strategies, the impact of basal p53 expression was studied in detail. Of general importance, we were able to demonstrate that not only protein regulations after p53-activating stimuli but also the p53 status of the tumor cell impacts apoptosis sensitivity both during extrinsic and intrinsic apoptosis induction.

The heterogeneous impact of p53 on cell death induction has attracted much notice during the recent years and further contributed to the complex and so far only rudimentary knowledge of p53 action. Formerly, p53 was thought to act primarily as a transcription factor and that the activity of p53 in wild-type conformation is mainly pro-apoptotic while certain mutants reduce the sensitivity towards apoptotic stimuli. Besides its transcriptional activating function, p53 was also proven for its repression of the transcription of anti-apoptotic proteins like Bcl-2 [25]. The recognition, that mutant p53 retains at least partial pro-apoptotic activities and that p53 in its non-mutated form can inhibit cell death induction, further contributed to the complexity [7,13,16,17]. Thereby, p53 activation within the identical cell can result in apoptosis promotion or inhibition depending on the stimuli and the experimental setting [2-4]. The lack of knowledge is evident, as p53 can also regulate protein expression independent from the direct transcriptional activity i.e. by post-transcriptional regulations and can directly activate the apoptotic machinery at the mitochondrial level [13,26-31]. We suggest that the impact of baseline p53 expression on the apoptosis inducing capacity of single agents and drug combinations should be studied in detail due to the major impact on extrinsic and intrinsic apoptosis sensitivity detected here. Current preclinical and phase I-III studies testing strategies to activate or modulate p53 functions should consider p53 functions independent from the classical view of protein or cell cycle regulations [8,9,30].

Specific p53 gene mutations displayed a variety of oncogenic properties mostly referred to as gain-of-function. These were mainly categorized according to the properties including tumor formation, augmented tumor cell growth, transformation, invasiveness, metastasis formation and inhibition of DNA repair and differed depending on the specific p53 mutation [14,15,32]. The data presented here clearly indicate a further dimension of complexity as wild-type p53 can act in a pro-apoptotic manner when the intrinsic cell death cascade is activated while it can reduce apoptosis sensitivity via the extrinsic signaling cascade in the identical tumor cell.

We had described before, that stimulation with TRAIL can result in four different net effects, namely cell death induction in type A cells, no effect on type 0 cells, simultaneous induction of apoptosis and proliferation in type AP cells and selective induction of proliferation in type P cells [33]. The heterogeneous responses described before probably reflect the diversity of each individual tumor and account for the complexity that has to be taken into account when the individual tumor cell is treated at its best. In line, the data presented in this study suggest that the cell death inducing capacity of TRAIL is regulated at many different steps and that besides the proper DISC formation, p53 itself has a major impact on the TRAIL efficacy. While for the proper DISC assembly, the presence and balance of the DISC members is critical, the p53 impact seems to be much more complex as it is not caused by the regulation of classical p53 target proteins, the cell cycle or spontaneous growth [1,33-35].

In summary, future preclinical and clinical studies investigating the implementation of the death inducing ligand TRAIL in clinical combination therapy protocols should not only take into account the critical signaling steps of TRAIL cell death induction but the impact of p53 status of the individual tumor cell on TRAIL sensitivity. The data presented here clearly indicate, that the restoration of p53 function is not always beneficial. Therefore, both strategies, restoration of p53 functionality and inhibition of p53 can be beneficial depending on the individual tumor. We suggest further evaluation of the therapeutic potential of in vitro drug sensitivity testing within in a similar experimental setting as described here. The results from the in vitro testing to predict the impact of p53 on apoptosis induction should be taken to determine the personalized in vivo treatment and to test the superiority of this approach in comparison to the standard protocol.

## Conclusions

The data presented add substantially to the knowledge of p53 functionality. p53 in wild-type status has long been thought to act as a tumor suppressor. Many studies have been performed to take advantage of the activation and restoration of wild-type p53 function which has proven beneficial in many different experimental settings. During the recent years, the complexity of p53 function has become evident. Unfortunately, at least under certain circumstances the advantage can turn into a disadvantage. The molecular data presented here prove that not only p53 activation but the baseline presence of p53 can impact cell death induction. The detailed analyses of p53 in wild-type conformation and of frequent p53 mutations disclose the complexity and the heterogeneous impact on extrinsic and intrinsic apoptosis induction. Surprisingly, even wild-type p53 status can act in an anti-apoptotic manner. The results presented highlight the need for the gain of knowledge and for the consideration of p53 function within the particular context and for the individual tumor to optimize therapy efficacy.

## Methods

### Materials

TRAIL was prepared as described recently [36]. Alternatively, TRAIL without any modification was obtained from Pepro Tech (Hamburg, Germany) and rendered identical results (data not shown). All further reagents were obtained from Sigma (St. Louis, MO).

For Western Blot, the following antibodies were used: anti-FADD, anti-FLIP and anti-XIAP from BD Biosciences (Franklin Lakes, NJ), anti-Bcl-xL, anti-Bid, anti-cIAP-1 and anti-PUMA from Cell Signaling; anti-Bak, anti-Bax, anti-Bcl-2, anti-cIAP-2, anti-Mcl-1 and anti-p53 from Santa Cruz (Santa Cruz, CA); anti GAPDH from Thermo Fisher (Waltham, MA) and anti NOXA from Calbiochem (San Diego, CA). For flow cytometric determination of TRAIL surface receptor expression, anti-DR4 and anti-DR5 were obtained from AXXORA (Lörrach, Germany) and anti-IgG conjugated to Alx647 from Life Technologies (Darmstadt, Germany).

### Cell lines, xenograft ALL cells and transfection experiments

HCT116, RKO and SW48 p53 +/+ and p53- / - cells were obtained from B. Vogelstein (Johns Hopkins University, Baltimore, MD). All further cell lines were obtained from DSMZ (Braunschweig, Germany) and maintained as described [2-4,37,38]. For leukemic cell line experiments, cells were seeded at 0,25 × 10^6^/ml, for stimulations with solid tumor cells at 0,05 × 10^6^/ml and incubated with TRAIL for 48 hours.

Informed consent was obtained from all patients in written form and studies were approved by the ethical committee of the medical faculty of the Ludwig Maximilians University Munich (LMU 068-08) and the children’s hospital of the TU Munich (TU 2115/08). Animal work was approved by the Regierung von Oberbayern (55.2-1-54-2531-2-07). The xenograft mouse model and engraftment, amplification, isolation and standardized procedures of siRNA interference and in vitro stimulation have been described in detail recently [3,4,21]. For the knockdown of p53, siRNA p53 (5^′^- GGGUUAGUUUACAAUCAGC -3^′^) was obtained from Ambion (Austin, TX) and as control All Star negative control siRNA from Qiagen (Hilden, Germany). The p53 status of the xenograft cells was determined using next-generation sequencing as described before [39].

Transfection experiments in cell lines where performed using lipofection or lentiviral transduction as described recently [2,3,38]. pCDH p53 constructs were generated by the insertion of p53 in wildtype status into the pCDH plasmid modified as described recently [34] and site specific mutations were generated by QuikChange II site-directed mutagenesis PCR kit from Agilent (Santa Clara, CA). Five different point mutations were generated by site-directed mutagenesis PCR at codon 143, 175, 248, 249 or 273. Shield-1 (Clontech, Saint-Germain-En-Laye, France) was used at 0,3 μM. shRNA p53 and corresponding mock sequences, constructs and protocols for transfection were previously described in detail [2,3,38]. DR4 and DR5 cDNAs obtained from imaGENES GmbH (Berlin, Germany) were cloned into pcDNA3.1 [36].

### Apoptosis assays, flow cytometry and Western blot analysis

For leukemia cell lines and xenograft ALL cells, forwardside scatter analysis was performed and verified using the recently described Annexin V – propidium iodid double staining [2]. For all adherent cell lines, cell death induction was determined using Nicoletti staining.

For the determination of TRAIL receptor surface expression, cells were washed in PBS followed by incubation with the primary antibody and by subsequent incubation with a dye-conjugation anti-IgG antibody conjugated to Alx647. The MFI (mean fluorescence intensity) was determined on a LSR II (BD Biosciences) using the Cell Quest Pro software version 3.2.1 (BD Biosciences) for data acquisition and FlowJo software version 8.3. (FlowJo, Ashland, OR) for data analyses.

Western Blot analysis was performed of total cellular lysates as described recently for cell lines and patient-derived leukemia cells [21,38]. Quantification of western blot analysis of primary samples by AIDA Image Analyzer (Raytest; Straubenhardt, Germany) had been described recently [3].

### Statistical analysis

TRAIL resistance was defined as cell death induction of <10% by 100 ng/ml TRAIL.

All data are presented as the mean values of at least three independent experiments ± SEM unless otherwise stated. To test for significant differences, the paired t-test was applied to compare two groups; for multivariate analysis, one way RM ANOVA was used. Statistical significance was accepted with p < 0,05.

### Availability of supporting data

The data sets supporting the results of this article are included within the additional file 1.

## Competing interests

The authors declare that they have no competing interests.

## Authors’ contributions

FW, CB, MG, DMW and HE performed experiments, FW and HE analyzed and interpreted the data and prepared the figures. HE designed the research and wrote the paper. IJ provided scientific support. All authors had a final approval of the manuscript.

## Supplementary Material

Additional file 1: Figure S1 TRAIL sensitivity not affected by TRAIL death receptor overexpression in SHEP cells. A,B)SHEP cells were transiently transfected with DR4 and DR5 expression plasmids. The change in DR4 (left panel) and DR5 (right panel) surface receptor expression was determined 36 hours after transfection as in Figure 1B (A) or cells were stimulated with TRAIL (100 ng/ml, B) for another 24 hours. Cell death induction, calculation of specific apoptosis, presentation of data and statistical analysis were performed as in Figure 1. *p < 0,05, one way RM ANOVA. NS = statistically not significant. **Figure S2.** Different impact of mutant p53 on extrinsic and intrinsic cell death induction in SHEP cells. A,B)pCDH constructs containing the different p53 variants were induced in SHEP cells as in Figure 2. SHEP cells were stimulated with doxorubicin (100 ng/ml, A) or TRAIL (100 ng/ml, B) for 48 hours. Cell death induction, calculation of specific apoptosis, presentation of data and statistical analysis were performed as in Figure 2. *p < 0,05, one way RM ANOVA. NS = statistically not significant. **Figure S3.** The heterogenous impact of p53 on extrinsic cell death induction in tumor cell lines. A-C)n = 8 pairs of cell lines with baseline p53 expression and downregulated p53 by RNA interference against p53, somatic knockout of p53 (SW48) or after transfection with the pCDH p53 wt expression plasmid (H1299) into p53 negative cells were separated according to their TRAIL response as in Figure 3A. TRAIL sensitivity in the presence of p53 was classified as augmented (A), unchanged (B) or reduced (C) efficacy. Stimulation with TRAIL, cell death induction, calculation of specific apoptosis, Western Blot analysis, presentation of data and statistical analysis were performed as in Figure 3. *p < 0,05, paired t-test. NS = statistically not significant. **Figure S4.** p53 knockdown by RNA interference in xenografted ALL cells. ALL-10S, ALL-54 and ALL-177 xenograft cells from Figure 4A were transfected with siRNA against p53. 48 hours later, Western Blot analysis was performed to prove knockdown efficiency. To enable the quantification of RNA interference, the p53 expression was investigated standardized to the expression level of GAPDH. The band density was analyzed using AIDA Image Analyzer and the relative expression level of p53 was calculated as (sample expression of p53 / sample expression of GAPDH) for sicontrol and sip53 transfected cells as described recently [21]. The reduction of p53 expression in sip53 transfected cells was calculated as [(relative p53 expression in sip53 cells / relative p53 expression in sicontrol cells) minus 1]. Click here for file
