# Supplementary material for: Impact of the p53 status of tumor cells on extrinsic and intrinsic apoptosis signaling
Source: Cell Commun Signal. 2013 Apr 17;11:27. doi: 10.1186/1478-811X-11-27 (PMC3641951; doi:10.1186/1478-811X-11-27)
Supplement: Additional file 1: Figure S1 — TRAIL sensitivity not affected by TRAIL death receptor overexpression in SHEP cells. A,B)SHEP cells were transiently transfected with DR4 and DR5 expression plasmids. The change in DR4 (left panel) and DR5 (right panel) surface receptor expression was determined 36 hours after transfection as in Figure 1B (A) or cells were stimulated with TRAIL (100 ng/ml, B) for another 24 hours. Cell death induction, calculation of specific apoptosis, presentation of data and statistical analysis were performed as in Figure 1. *p < 0,05, one way RM ANOVA. NS = statistically not significant. Figure S2. Different impact of mutant p53 on extrinsic and intrinsic cell death induction in SHEP cells. A,B)pCDH constructs containing the different p53 variants were induced in SHEP cells as in Figure 2. SHEP cells were stimulated with doxorubicin (100 ng/ml, A) or TRAIL (100 ng/ml, B) for 48 hours. Cell death induction, calculation of specific apoptosis, presentation of data and statistical analysis were performed as in Figure 2. *p < 0,05, one way RM ANOVA. NS = statistically not significant. Figure S3. The heterogenous impact of p53 on extrinsic cell death induction in tumor cell lines. A-C)n = 8 pairs of cell lines with baseline p53 expression and downregulated p53 by RNA interference against p53, somatic knockout of p53 (SW48) or after transfection with the pCDH p53 wt expression plasmid (H1299) into p53 negative cells were separated according to their TRAIL response as in Figure 3A. TRAIL sensitivity in the presence of p53 was classified as augmented (A), unchanged (B) or reduced (C) efficacy. Stimulation with TRAIL, cell death induction, calculation of specific apoptosis, Western Blot analysis, presentation of data and statistical analysis were performed as in Figure 3. *p < 0,05, paired t-test. NS = statistically not significant. Figure S4. p53 knockdown by RNA interference in xenografted ALL cells. ALL-10S, ALL-54 and ALL-177 xenograft cells from Figure 4A were transfected with siRNA [file 1478-811X-11-27-S1.pptx]

## Slide 1
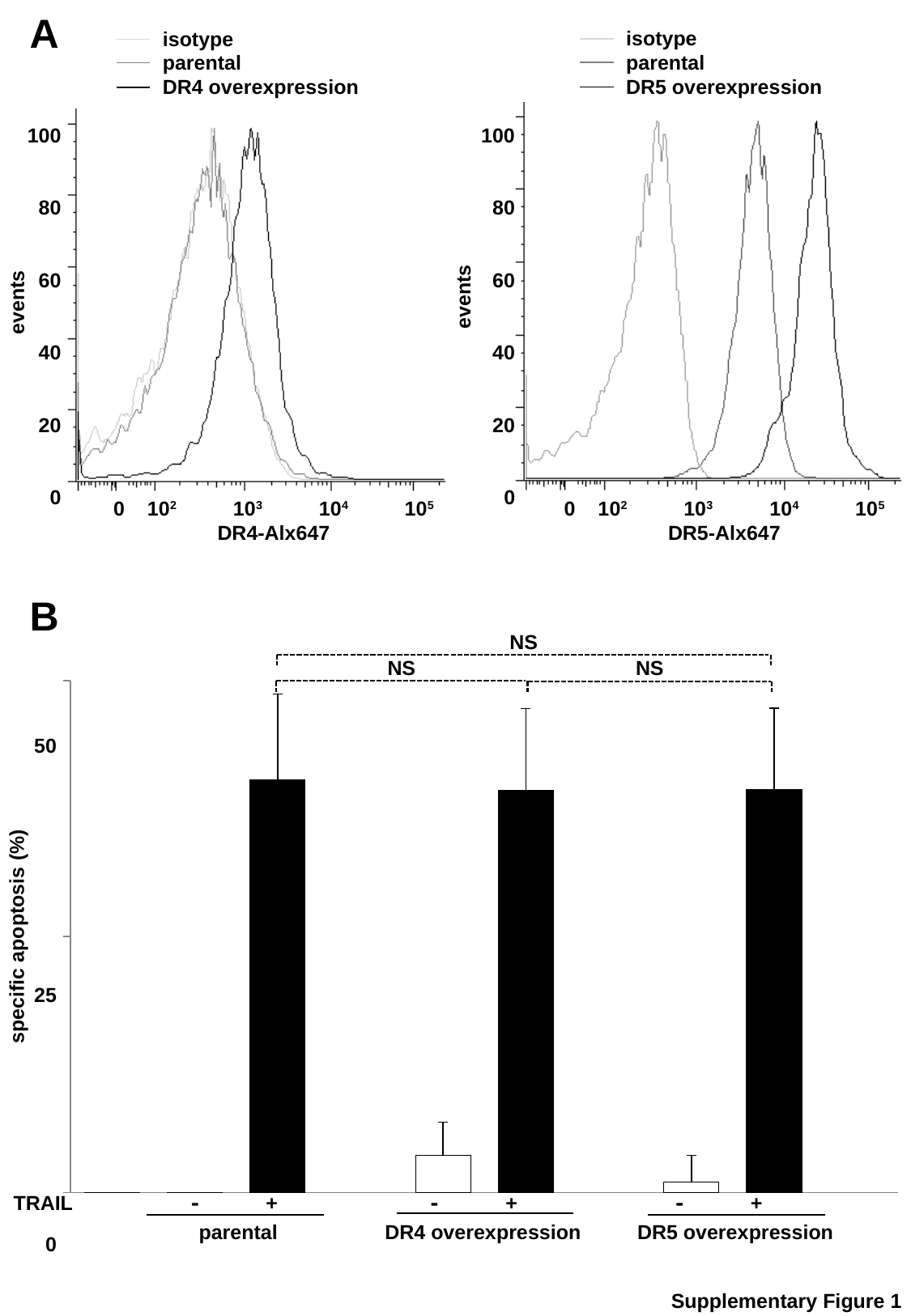

A
B
isotype
parental
DR5 overexpression
isotype
parental
DR4 overexpression
100
80
60
40
20
0
100
80
60
40
20
0
events
events
0 102 103 104 105
DR4-Alx647
0 102 103 104 105
DR5-Alx647
50
25
0
NS
NS
NS
### Chart
| Category | mean |
|---|---|
| test | 0.0 |
| co | 0.005 |
| tr | 40.33 |
| | None |
| DR4 | 3.67 |
| TR | 39.3 |
| | None |
| DR5 | 1.0 |
| TR | 39.33 |specific apoptosis (%)
TRAIL − + − + − + ­
parental DR4 overexpression DR5 overexpression
Supplementary Figure 1

## Slide 2
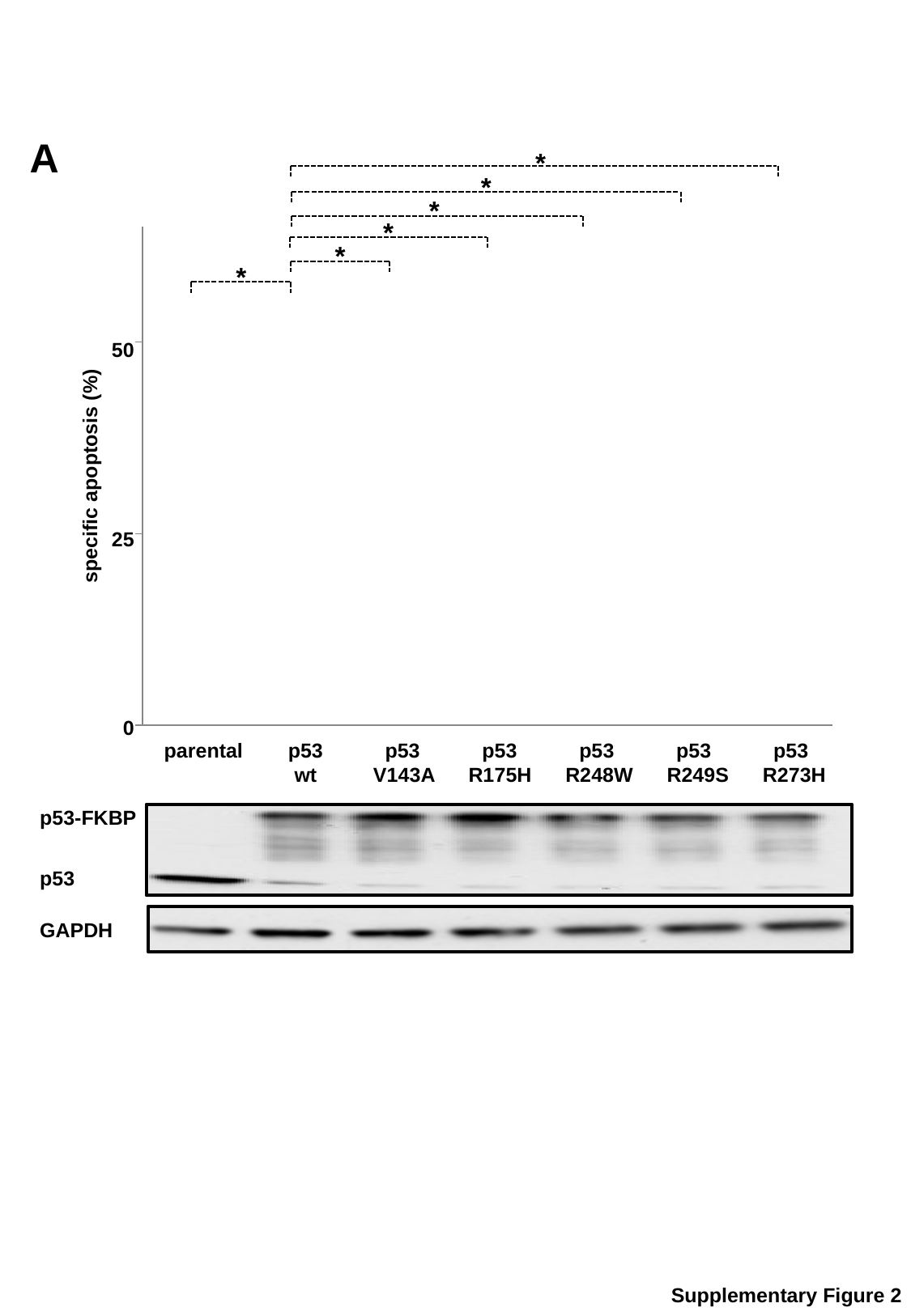

A
*
*
*
*
### Chart
| Category | mean |
|---|---|
| par | 0.167 |
| wt | 0.45 |
| 143 | 0.09 |
| 175 | 0.106 |
| 248 | 0.13 |
| 249 | 0.1 |
| 273 | 0.146 |*
*
50
25
0
specific apoptosis (%)
 parental p53 p53 p53 p53 p53 p53
 wt V143A R175H R248W R249S R273H
p53-FKBP
p53
GAPDH
Supplementary Figure 2

## Slide 3
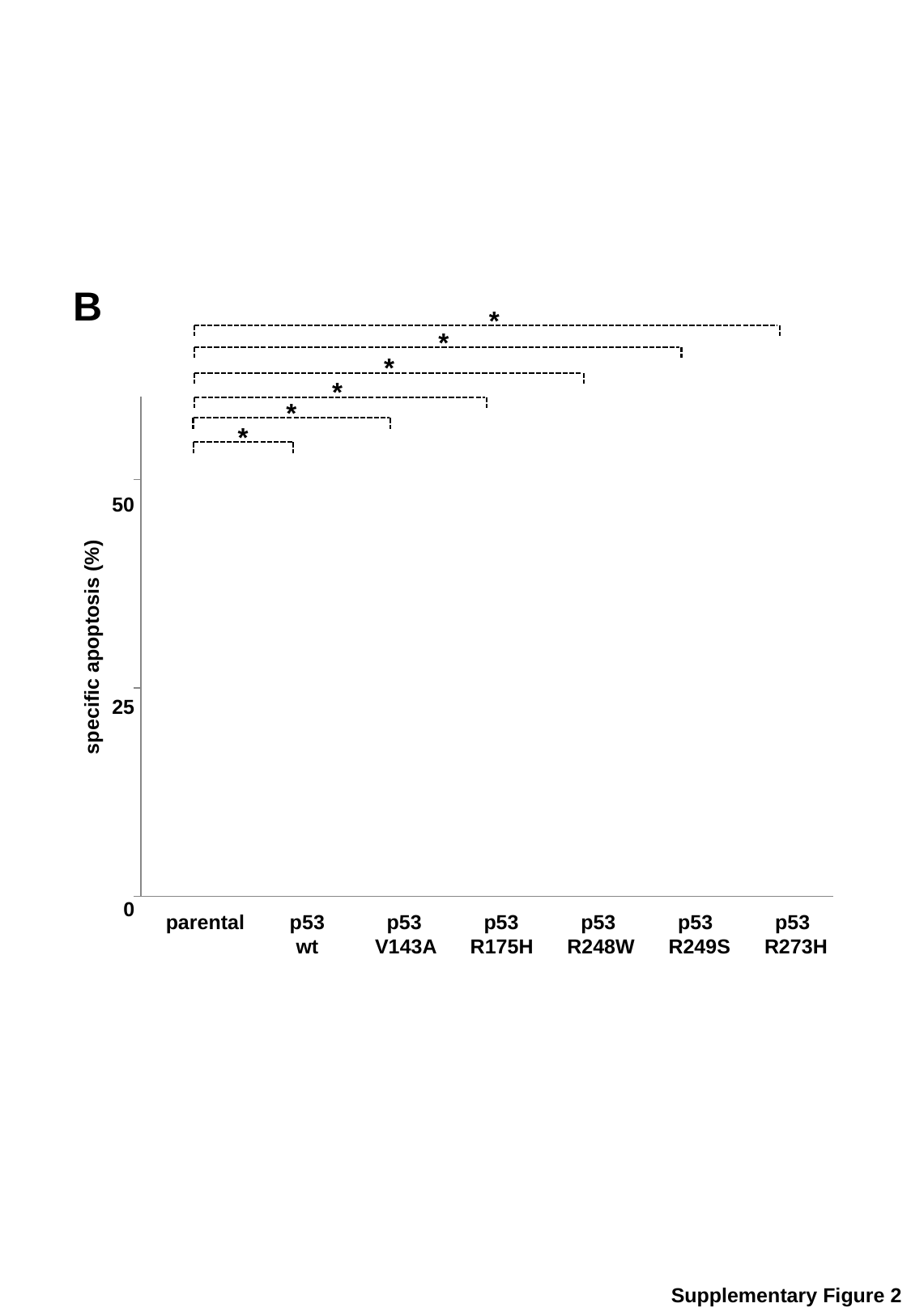

B
*
*
*
*
### Chart
| Category | mean |
|---|---|
| par | 0.46 |
| wt | 0.28 |
| 143 | 0.35 |
| 175 | 0.37 |
| 248 | 0.27 |
| 249 | 0.3 |
| 273 | 0.35 |*
50
25
0
*
specific apoptosis (%)
 parental p53 p53 p53 p53 p53 p53
 wt V143A R175H R248W R249S R273H
Supplementary Figure 2

## Slide 4
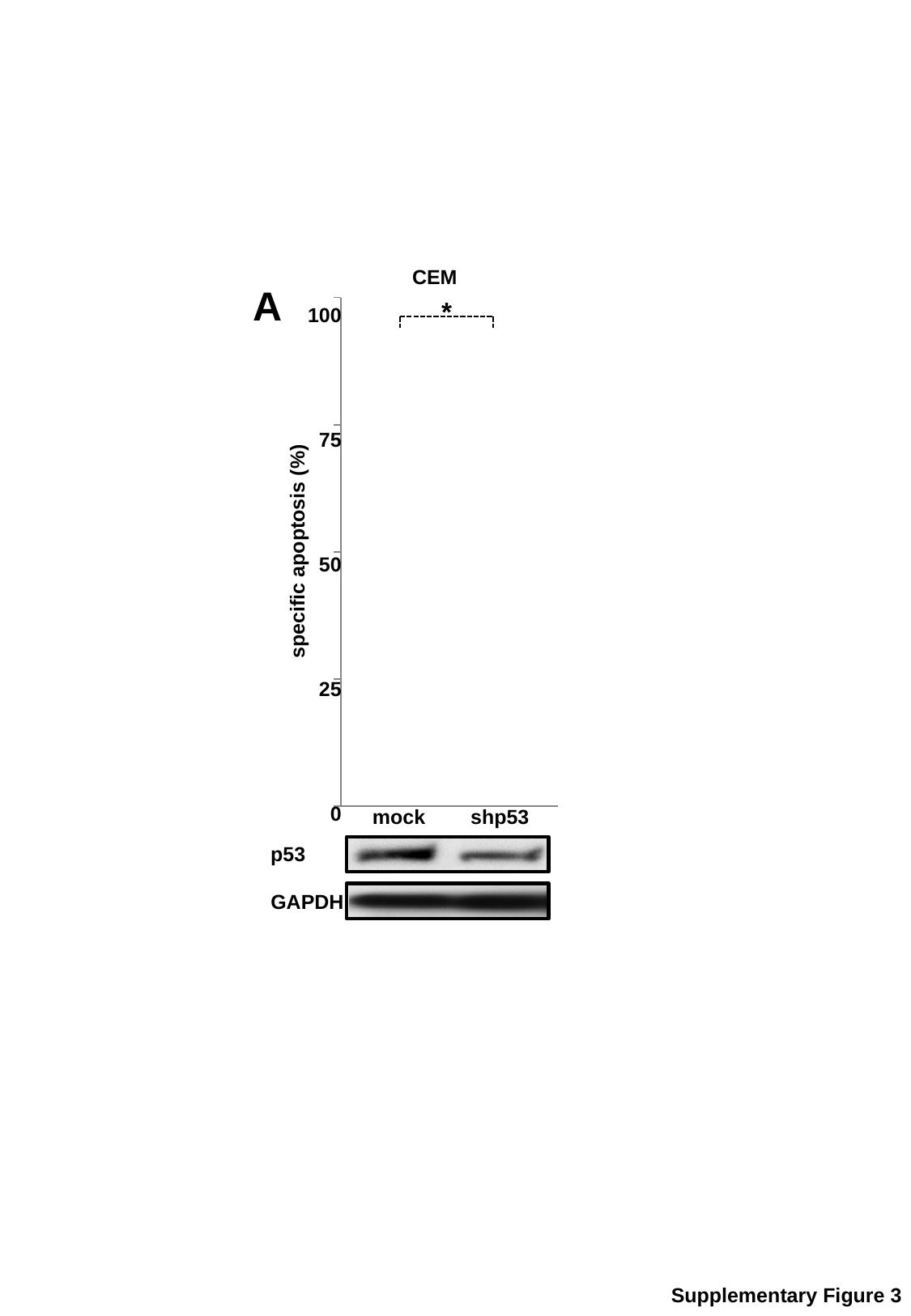

100
75
50
25
0
### Chart
| Category | mean |
|---|---|
| TRAIL | 0.897 |
| MTX | 0.64 |*
specific apoptosis (%)
mock shp53
p53
GAPDH
CEM
A
Supplementary Figure 3

## Slide 5
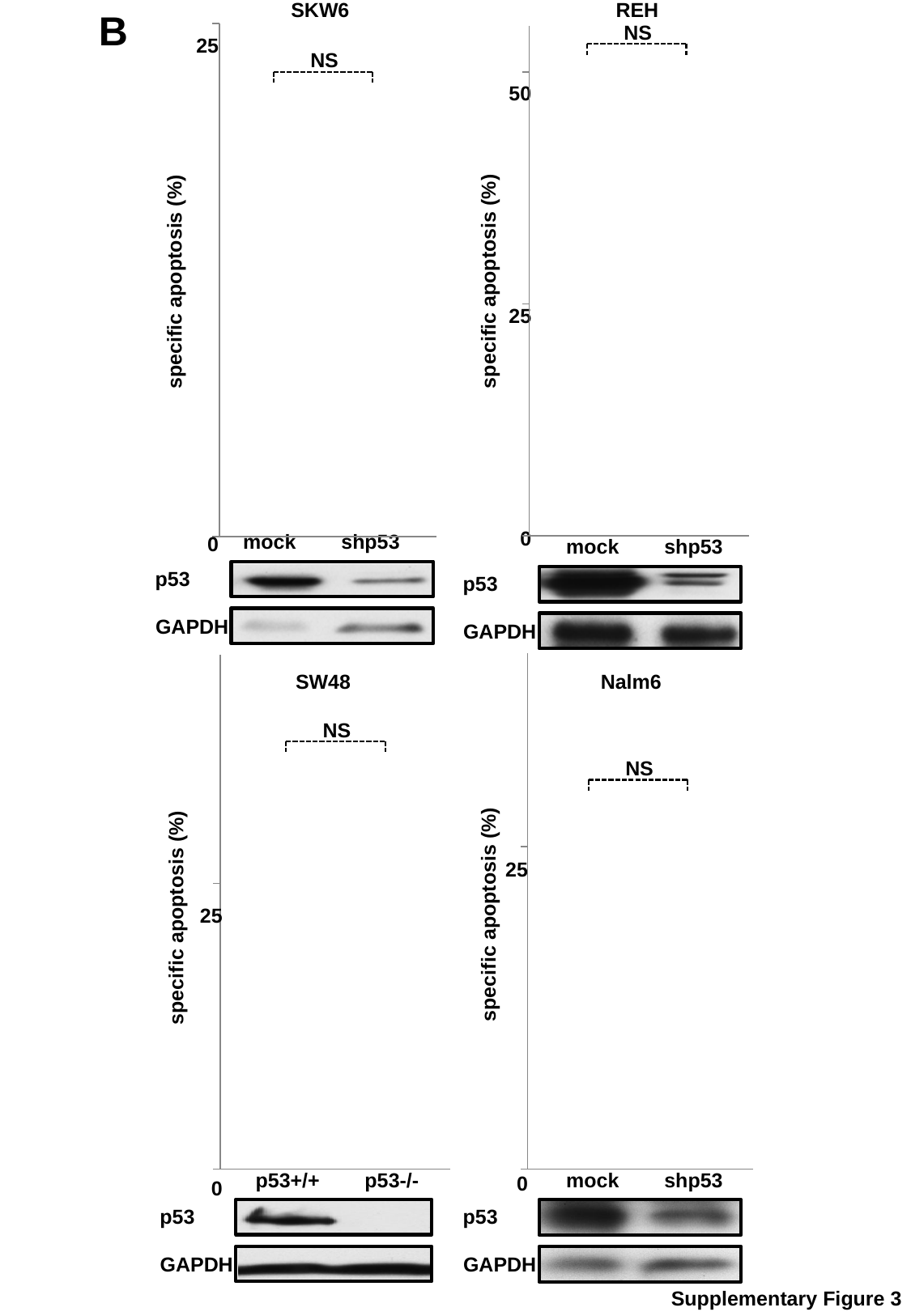

25
0
SKW6
REH
### Chart
| Category | mean |
|---|---|
| TRAIL | 0.13 |
| MTX | 0.16 |NS
### Chart
| Category | mean |
|---|---|
| TRAIL | 0.42 |
| MTX | 0.39 |50
25
0
specific apoptosis (%)
mock shp53
p53
GAPDH
NS
specific apoptosis (%)
mock shp53
p53
GAPDH
B
25
0
### Chart
| Category | mean |
|---|---|
| TRAIL | 0.18 |
| MTX | 0.22 |
### Chart
| Category | mean |
|---|---|
| TRAIL | 0.22 |
| MTX | 0.28 |
25
0
SW48
Nalm6
NS
NS
specific apoptosis (%)
specific apoptosis (%)
p53+/+ p53-/-
mock shp53
p53
p53
GAPDH
GAPDH
Supplementary Figure 3

## Slide 6
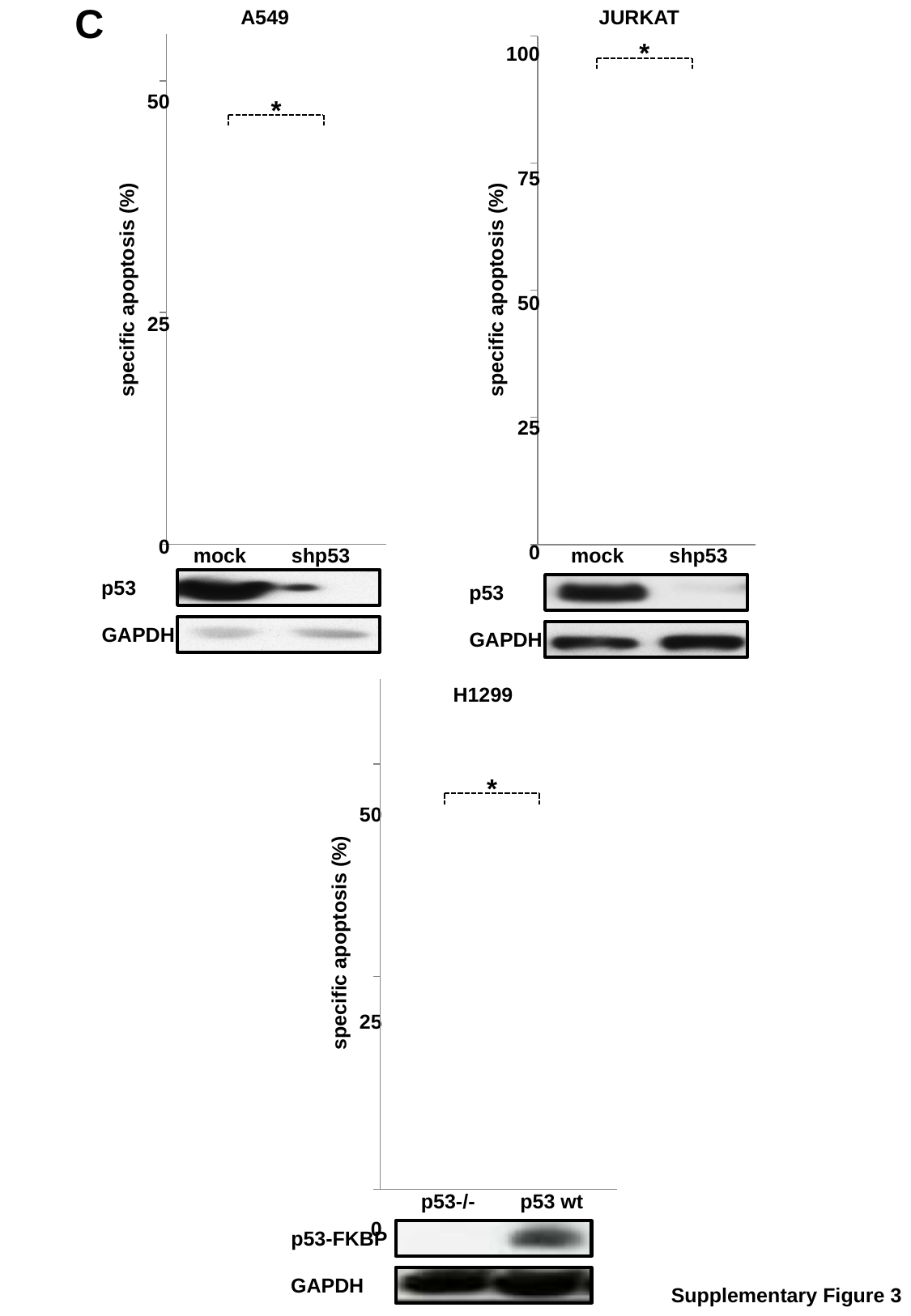

C
100
75
50
25
0
A549
JURKAT
### Chart
| Category | mean |
|---|---|
| TRAIL | 0.15 |
| MTX | 0.373 |
### Chart
| Category | mean |
|---|---|
| TRAIL | 0.62 |
| MTX | 0.9 |*
50
25
0
*
specific apoptosis (%)
specific apoptosis (%)
mock shp53
mock shp53
p53
p53
GAPDH
GAPDH
50
25
0
### Chart
| Category | mean |
|---|---|
| TRAIL | 0.34 |
| MTX | 0.05 |*
specific apoptosis (%)
 p53-/- p53 wt
p53-FKBP
GAPDH
H1299
Supplementary Figure 3

## Slide 7
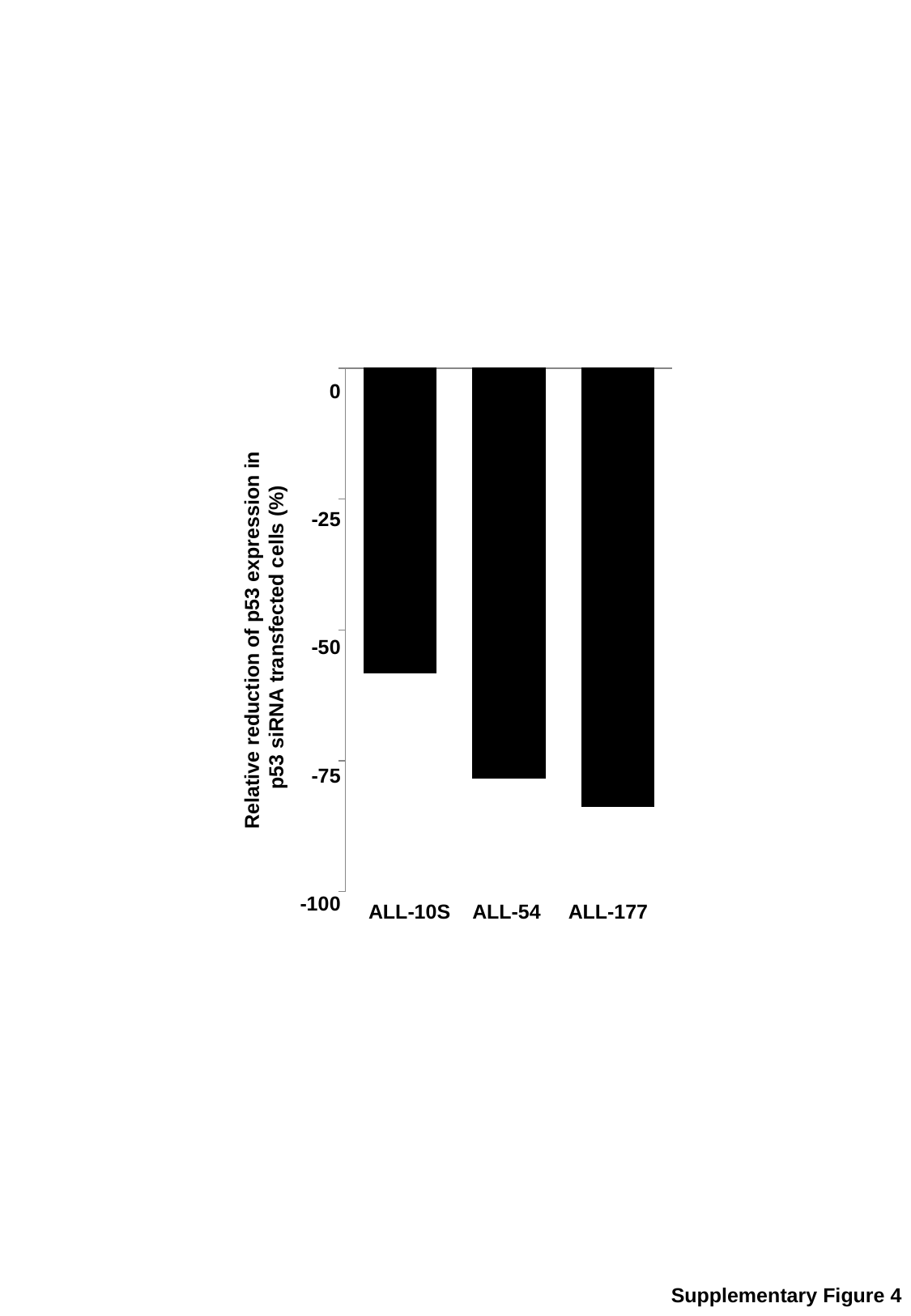

0
-25
-50
-75
-100
### Chart
| Category | relative reduction p53 |
|---|---|
| 10S | -0.5821305527694016 |
| 54 | -0.7837113383072768 |
| 177 | -0.8374531191503984 |Relative reduction of p53 expression in
p53 siRNA transfected cells (%)
 ALL-10S ALL-54 ALL-177
Supplementary Figure 4
